# Derby Day and "Recreative Betting."

**Published:** 1912-06-08

**Authors:** 


### DERBY DAY AND "RECREATIVE BETTING."

The psychiatry of sport has been discussed by many authors from Laurence Sterne, who treated the subject in his accustomed racy style, to Lombroso and Krafft-Ebing, who brought to the debate a wealth of scientific nomenclature which consternates more than it impresses. The sum and upshot of it all is to the effect that men (and women) take their pleasures excitedly or quietly according to their psychological condition—a conclusion which no one needed the impressive pronouncement of a scientist to confirm. The variety and diversity of physiognomy on that early Victorian masterpiece by Frith depicting the crowd on Derby Day are interesting to all of us who desire to know in how far sport and the cumulative effects of social environment have changed the character of the nation. To moderns the faces in that picture are unreal, just as grotesque as are some of the dresses.

In what way, then, does sport, and especially such typically national events as a cup-tie or the Derby, influence national life, character, and that individually vague but collectively very distinctive entity which we call national stamina? Open-air life, if only for a day, absence of care, gaiety, frank enjoyment, all of them, if only for a term of so many hours once or twice during every twelvemonth, inevitably benefit those who indulge in them. And the vast majority of those who ride Epsomwards, more so, perhaps, than those who go to witness a cup-tie, go to spend a holiday in the open. The race itself, notwithstanding the "prophet" or the "welsher," is a secondary consideration. To them the day is as much a holiday as is Whit Monday or Boxing Day. It is a time of recreation such as no nation or community can afford to be without.

Naturally there is another side to the question. To many Derby Day is a day of mental stress and nervous excitement. Indeed, the spasm of nervous stimulation which it causes runs through the whole of the British Empire. The attraction of the sweepstake is universal, and white men are no less liable to the temporary excitement that it induces than the Chinese. But it is doubtful if the European at least reacts to the stimulus in the same manner as do the Oriental peoples. It is true enough, as psychiatrists know, that to some persons the emotional effects of expectation are supremely detrimental, but these are obviously exceptional natures which probably would react abnormally to any stimulus. The fact remains that the mass of the people does not appear to be psychologically deteriorated by the side issues of Derby Day. In the list of causative factors of insanity and mental derangement, it is a striking fact that sport and what may be called recreative betting occupy a position which is utterly insignificant when compared with Stock Exchange excitement or, still worse, religious stress.

This is very gratifying, for already sport, and especially racing, have to withstand attacks which depend, so far as the gravamen of the indictment is concerned, upon a certain pseudo-scientific line of reasoning which premises theoretical postulates and concludes generalities on an elench. Appeals are made to the racing mania which has been noticed in some Eastern colonies, where native princes spend large sums on the sport. Against this, quite properly, may be set the equally absurd fashion of mosque-building, which in the same States probably consumes more money than the purchase and upkeep of a racing stud. On such issues the question cannot be discussed. If we are to have any useful conclusions let us have facts which will tend to show whether or not indulgence in sport, as obtains at present in all civilised countries, but especially in this country, which has made Derby Day into a national and imperial institution, is physically and psychologically injurious to the normal section of the people. To conclude such injury from the effects of its stimulus upon the small section of the abnormal in the community is to employ the "argument of Tenterden steeple"—a method of reasoning which to a professional man must be as distasteful as Gil Blas's criticism was to the Archbishop of Toledo.

